# Early Evolutionary Selection of NAD Biosynthesis Pathway in Bacteria

**DOI:** 10.3390/metabo12070569

**Published:** 2022-06-21

**Authors:** Suraj Sharma, Yin-Chen Hsieh, Jörn Dietze, Mathias Bockwoldt, Øyvind Strømland, Mathias Ziegler, Ines Heiland

**Affiliations:** 1 Department of Arctic and Marine Biology, Faculty of Biosciences, Fisheries and Economics, UiT The Arctic University of Norway, 9037 Tromsø, Norway; suraj.sharma@uit.no (S.S.); hsieh.y.chen@uit.no (Y.-C.H.); jorn.dietze@uit.no (J.D.); 2 Research Centre for Arctic Petroleum Exploration (ARCEx), Department of Geosciences, UiT The Arctic University of Norway, 9037 Tromsø, Norway; mathias.bockwoldt@uit.no; 3 Department of Biomedicine, University of Bergen, 5020 Bergen, Norway; oyvind.stromland@uib.no (Ø.S.); mathias.ziegler@uib.no (M.Z.); 4 Department of Clinical Medicine, University of Bergen, 5020 Bergen, Norway

**Keywords:** NAD biosynthesis, metabolic modelling, kinetic models, phylogenetic analysis

## Abstract

Bacteria use two alternative pathways to synthesize nicotinamide adenine dinucleotide (NAD) from nicotinamide (Nam). A short, two-step route proceeds through nicotinamide mononucleotide (NMN) formation, whereas the other pathway, a four-step route, includes the deamidation of Nam and the reamidation of nicotinic acid adenine dinucleotide (NAAD) to NAD. In addition to having twice as many enzymatic steps, the four-step route appears energetically unfavourable, because the amidation of NAAD includes the cleavage of ATP to AMP. Therefore, it is surprising that this pathway is prevalent not only in bacteria but also in yeast and plants. Here, we demonstrate that the considerably higher chemical stability of the deamidated intermediates, compared with their amidated counterparts, might compensate for the additional energy expenditure, at least at elevated temperatures. Moreover, comprehensive bioinformatics analyses of the available >6000 bacterial genomes indicate that an early selection of one or the other pathway occurred. The mathematical modelling of the NAD pathway dynamics supports this hypothesis, as there appear to be no advantages in having both pathways.

## 1. Introduction

**Figure 1:**
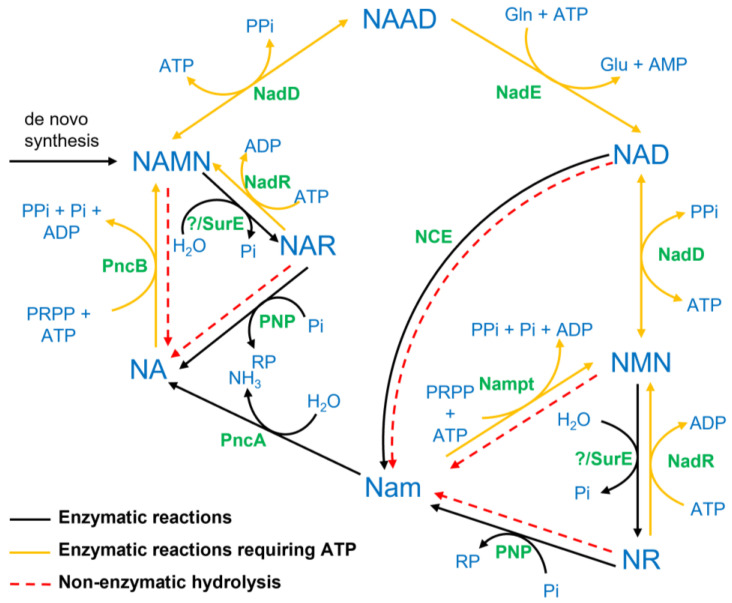
Schematic representation of reactions involved in the biosynthesis of NAD in different organisms. Metabolite names are written in bold blue letters, while the enzyme names are denoted in green colour. The abbreviation NCE (NAD-consuming enzymes) represents all enzymes catalysing signalling reactions consuming NAD. The bold black lines represent enzyme-catalysed reactions, whereas the dotted red lines denote non-enzymatic hydrolysis, mainly occurring at high temperatures. Bold yellow lines are used to denote reactions that require ATP. Whether the bacterial 5′-nucleotidase SurE accepts NMN/NAMN as substrate is unknown; a homologue to the mammalian NT5 could not be identified in bacteria. NAD, Nam adenine dinucleotide; NMN, Nam mononucleotide; NR, Nam riboside; NA, nicotinic acid; NAR, NA riboside; NAMN, NA mononucleotide; NAAD, NA adenine dinucleotide; NadD, NA/NMN adenylyltransferase; Nampt, Nam phosphoribosyltransferase; NadR, NA/Nam riboside kinase; SurE, 5′-nucleotidase; PNP, purine nucleoside phosphorylase; PncA, nicotinamidase; PncB, NA phosphoribosyltransferase; NadE, NAD synthase.

NAD is an essential cofactor in all organisms. It serves as an electron acceptor for a multitude of redox reactions and is involved in a large number of signalling reactions. In contrast to the reversible interconversion in redox reactions, signalling reactions consume NAD and release nicotinamide (Nam). This needs to be compensated by a constant synthesis of NAD. Two different pathways exist to synthesize NAD from Nam [1,2]. The more common pathway in yeast, plants and bacteria starts with the deamidation of Nam by the bacterial nicotinamidase (PncA) producing nicotinic acid (NA). The Preiss–Handler pathway is employed to convert NA into NAD in a three-step process starting with NA phosphoribosyltransferase (PncB), converting NA into its mononucleotide, NAMN [3]. NAMN is then converted into the dinucleotide NAAD by NA/Nam mononucleotide adenylyltransferase (NadD), which is present in all organisms [4]. The last step is the amidation of NAAD to NAD, catalysed by NAD synthase (NadE). The alternative pathway dominant in higher vertebrates but also present in bacteria [1] converts Nam directly into its mononucleotide, NMN, through nicotinamide phosphoribosyltransferase (Nampt), followed by adenylation through NadD. In addition to synthesis from Nam, NAD can be de novo synthesized from aspartate or tryptophan. An overview of the NAD biosynthesis pathways is shown in Figure 1.

The four-step process that uses PncA requires reamidation to convert NAAD into NAD. This reaction requires ATP releasing AMP [5]; therefore, it is energetically expensive. Thus, the four-step NAD biosynthesis via PncA and NadE requires more ATP than the two-step process that uses Nampt. It is, therefore, surprising that this pathway is dominant in bacteria, yeast and plants. To better understand the evolutionary process that led to preferential selection in bacteria, we performed a detailed phylogenetic analysis of the two pathways focusing on the presence or absence of PncA and Nampt [1,2]. A detailed analysis of this distribution revealed that the selection of the pathways might have occurred based on habitat preferences with a predominant presence of PncA in extremophile and especially in thermophile organisms. We, therefore, measured the thermostability of different pathway intermediates and constructed a temperature-dependent mathematical model including the two alternative pathways of NAD biosynthesis. Model simulations suggest that despite the high thermolysis rates of Nam intermediates, NAD biosynthesis via PncA has no clear advantages over the Nampt pathway at high temperatures. The simulations furthermore indicated that the optimization of NAD biosynthesis indicates that there are no advantages in having both the PncA and the Nampt pathway.

## 2. Results

### 2.1. Early Evolutionary Selection of the PncA or the Nampt Pathway in Bacteria

**Figure 2:**
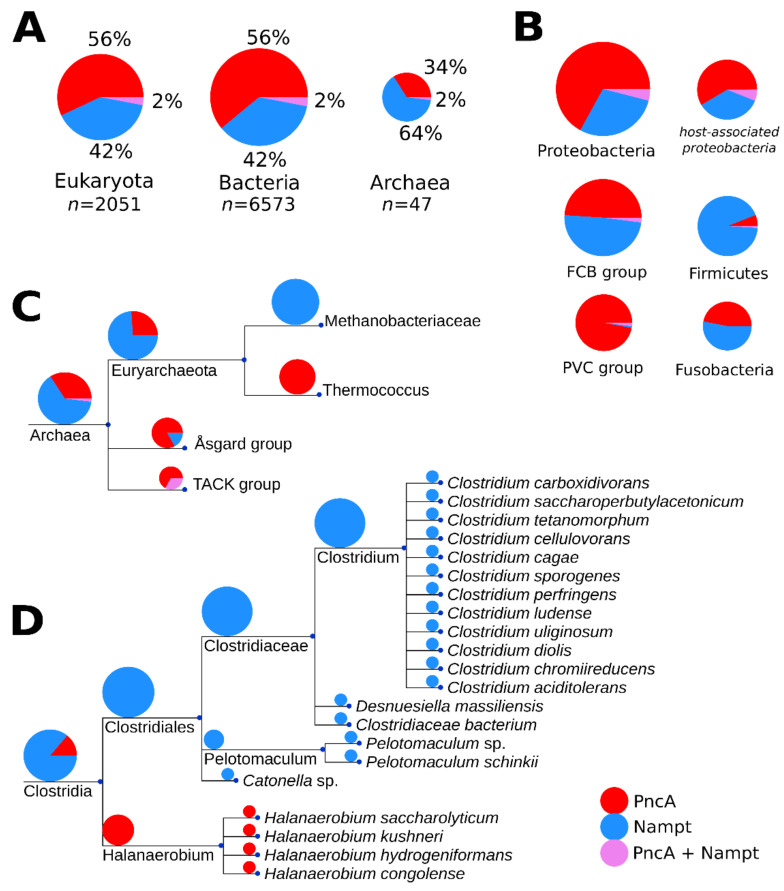
Phylogenetic distribution of PncA and Nampt among the three domains, with expansion into archaea and the bacterial taxa of clostridia. Panel (**A**) shows the total number of organisms (*n*) represented in the phylogenetic analysis, per domain, as well as their proportions as coloured in the following scheme (PncA, red; Nampt, blue; both PncA and Nampt, violet). The numeric proportion is written beside each segment of the pie chart. The pie chart sizes in each panel are scaled proportionally to the logarithm of the number of organisms represented in each group. In panel (**B**), the enzyme distribution in the largest bacterial clades is shown. Host-association patterns in the proteobacterial group are also shown as an additional pie chart to the right of the total proteobacterial pie chart. Panel (**C**) expands on the domain of archea, particularly to two clades within the phylum euryarchaeota that have presence of only PncA (Thermococcus) or Nampt (Methanobacteriaceae). Panel (**D**) expands on the bacterial class of clostridia, which is a member of the phylum firmicutes. Clostridia is one of several bacterial classes that exhibit a clear preference for PncA in extremophiles, based on our phylogenetic analyses. Branch lengths of the trees are arbitrary.

To better understand the prevalence of PncA over Nampt in bacteria, we performed a detailed phylogenetic analysis of the distribution of these enzymes in eukaryotes, bacteria and archaea in more than 8000 organisms (Figure 2A). A previous study of about 200 bacterial genomes described a scattered distribution [2]. In contrast, our phylogenetic analysis of more than 6000 bacteria and archaea detected a mutually exclusive presence of PncA and Nampt, with a clear separation between taxa containing either of the enzymes (Figure 2). This indicates that common ancestors likely harboured both enzymes, with pathway selection having occurred in early evolution.

In archaea, members of genus Thermococcus exclusively harbour PncA, whereas species within the Methanomada group exclusively encode Nampt. We also detected a predominant presence of PncA in thermophilic bacteria. In Clostridia (Figure 2D), for example, the two taxonomic groups show a clear separation between extremophile and non-extremophile organisms. Many of the non-extremophile organisms are pathogenic and/or are known to have vertebrates as hosts. A clear preference is difficult to identify as the habitat information is incomplete, and some organisms can be pathogenic but also live in other habitats such as soil.

Nevertheless, to obtain further insights into a potential role of host association in pathway selection, we used the available habitat information of proteobacteria from the Integrated Microbial Genomes (IMG) database [6,7] and matched organisms found in our phylogenetic analysis with those annotated as being associated with a mammalian host. Annotations for 3154 proteobacterial entries from the IMG were retrieved. Figure 2B shows the host-association patterns of Nampt and PncA within this proteobacterial group. The distribution appears to be similar as in bacteria overall, suggesting that there are no preferences for Nampt in mammalian-host-associated proteobacteria. Thus, host association does not appear to be a dominant selection criterium.

### 2.2. Higher Glycohydrolysis of Nam Pathway Intermediates at High Temperatures

**Figure 3:**
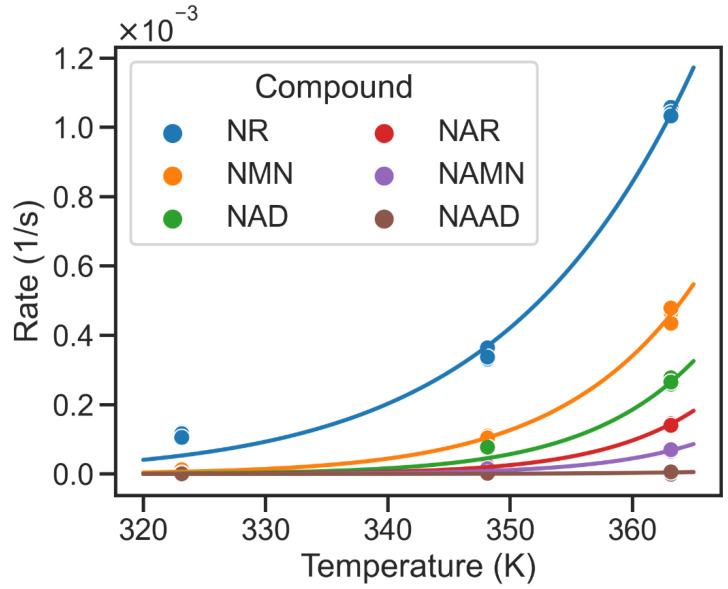
Temperature dependency of non-enzymatic hydrolysis of metabolic intermediates of NAD biosynthesis. Temperature on x-axis is reported in Kelvin (K). Hydrolysis rates are given per second (1/s). While the solid dots represent measured hydrolysis rates, the solid lines denote the temperature-dependent hydrolysis rates calculated using a fitted Arrhenius equation (cf. Table 1). NAAD shows the lowest hydrolysis rates, while NR exhibits the highest glycohydrolysis rate at 363.15 K.

NAD has been shown to undergo rapid glycohydrolysis at high pH values with Nam and ADP-ribose as products [8,9]. The predominance of PncA in thermophilic organisms may, therefore, be related to the higher chemical stability of acidic NAD biosynthetic intermediates. Therefore, we decided to further analyse the temperature-dependent characteristics of the NAD biosynthesis pathway. As data about the temperature-dependent stability of other intermediates of the NAD biosynthetic pathways were not available, we measured the rate of non-enzymatic hydrolysis of the relevant NAD biosynthesis intermediates at different temperatures in vitro (cf. Table 1). These measurements showed that Nam metabolites such as NMN and NR exhibited higher hydrolysis rates than NAD, especially at high temperatures, whereas the hydrolysis rates of NA intermediates were much lower with no detectable hydrolysis of NAAD (Figure 3) even at 363.15 K (90 °C). These observations suggest a potential advantage of the PncA/Preiss–Handler pathway over the shorter two-step pathway at a higher temperature.

### 2.3. PncA Pathway Is More Energy Demanding Even at High Temperatures

To analyse the potential effects of the non-enzymatic hydrolysis at high temperatures of nicotinamide intermediate on the efficiency of NAD biosynthesis, we developed a mathematical model of NAD biosynthesis using temperature-dependent rate laws. For the purpose, we integrated relevant enzymatic reactions and rate laws from our previously published NAD model (BioModels: MODEL1905220001; [1]). Additionally, to make the model temperature dependent, the reaction rates were scaled using the Arrhenius equation (cf. Equation (1)). As no measured activation energies were available for the enzymatic reactions, we set the activation energies such that the reactions were thermodynamically feasible at physiological conditions and had a Q10 within the range of 2–3, as previously conducted [10,11]. Further, we added non-enzymatic hydrolysis reactions using rates and activation energies determined by fitting the experimentally measured data (see Table 1) to the Arrhenius equation. The resulting model is based on a set of ordinary differential equations (ODEs) that can simulate temperature-dependent steady-state changes in the pathway intermediates of NAD biosynthesis via PncA and Nampt (for details see Supplementary Equations (S1)–(S8)).

To estimate the energy efficiency of the two alternative biosynthetic routes, we calculated the ratio between ATP consumption and NAD production. ATP consumption is defined as the total flux of ATP-consuming reactions (*J_ATP_*) and is caused by the following reactions catalysed by: Nampt, PncB, NadE, NadD and NadR. The amidation of NAAD uses one ATP molecule to produce NAD and one AMP. As two phosphorylation steps are required to convert AMP back to ATP, we represent the energy demand of the NadE catalysed reaction by multiplying its flux by 2. The total flux of ATP-consuming reactions is thus:

(1) $$J_{ATP}=J_{NAMPT}+J_{PNCB}+2\;\cdot \;J_{NADE}+J_{NADD}+J_{NADR}.$$

The synthesis of the mononucleotides through Nampt or PncB is non-stoichiometrically coupled to the hydrolysis of ATP owing to the autophosphorylation of a histidine residue, thereby increasing substrate affinity. For NAMPT, it has been estimated that about one ATP is consumed per catalytic cycle at an ATP concentration of 2–2.5 mM [12]. Given the highly similar reaction and enzyme structures, this ATP requirement can be assumed to be the same for both enzymes; therefore, it has most likely no or little influence on the efficiency of either pathway.

NAD production is attributed to the flux generated by NadE (*J_NADE_*) in the PncA pathway and NadD (*J_NADD_*) in the Nampt pathway converting NAAD and NMN to NAD, respectively:

(2) $$J_{NAD}=J_{NADE}+J_{NADD}$$

To investigate the efficiency of the two biosynthetic routes of NAD production, we assumed that the bacterial NAD biosynthetic pathway adjusts the concentration of metabolic enzymes to minimize the energy demand defined by the ratio of the total flux of ATP-consuming reactions (*J_ATP_*) to the NAD production flux (*J_NAD_*) given a range of free-NAD concentration. The optimization problem can, therefore, be represented as:

(3) $$\underset{E\in \left[1^{-10},100\right]}{min}\;\frac{J_{ATP}}{J_{NAD}}\;subject\;to:\;0.1<C_{NAD}<0.3\;B=\left\{x\vee 0.01\leq x\leq 100\right\}\;\underset{i}{\sum }E\leq 1000;i=1,\ldots ,n$$

This represents the value of the argument E (E=A∪B, where $A=\{E_{PNCA},E_{NAMPT}\}$ and $B=\left\{E_{PNCB},E_{NADD},E_{NADR},E_{PNP},E_{NADE},E_{NCE},E_{SURE}\right\}$) in the interval [1^−10^, 100] nM that minimizes objective function *J_ATP_*/*J_NAD_*, with the added constraints that the steady-state concentration of NAD is between 0.1 and 0.3 mM; the sum of concentrations of n metabolic enzymes is less than 1000 nM; and the subset B is the set of all elements x, such that x is in the interval [0.01, 100] nM. The first inequality constraint ensures the experimentally measured free-NAD concentrations in bacteria, whereas the latter two constrain the lower and upper limits on the chosen concentrations of metabolic enzymes. Optimization was performed using the Evolutionary Programming algorithm of COPASI 4.29 [13], with the number of generations and population size being set to 200 and 20, respectively. The selection of these optimization parameters was based on the convergence of the desired output within the tolerance range of 1 × 10^−6^.

**Figure 4:**
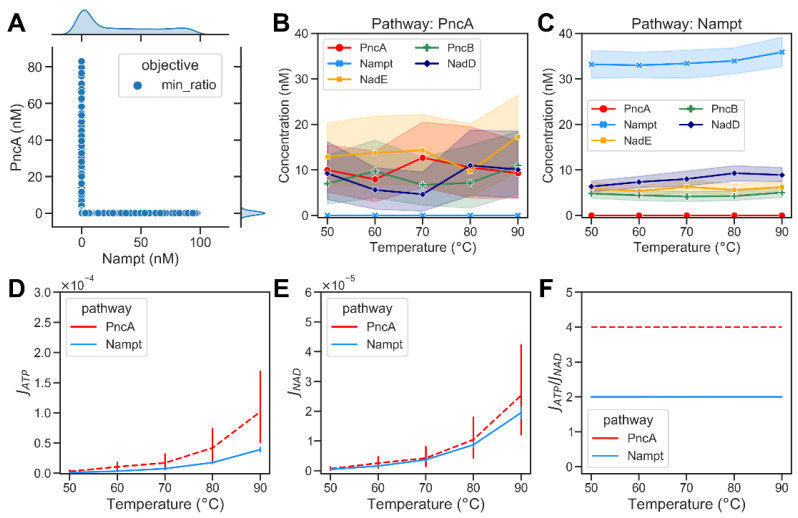
Optimization results of minimizing the energy demands of bacterial NAD biosynthetic pathways. (**A**) A scatter plot showing the optimized abundance of Nampt versus PncA when minimizing the ratio of ATP consumption to NAD production required to maintain the required NAD steady-state concentration ($0.1\;\mathrm{mM}<C_{NAD}<0.3\;\mathrm{mM}$). Each dot represents an optimisation result. The marginal plots show kernel density estimate plots that show the enzyme abundance distributions in the optimisation results. (**B**) Abundance of metabolic enzymes at different temperatures in the PncA pathway. (**C**) Optimized abundance of metabolic enzymes in Nampt pathway. (**D**) The ATP consumption flux, $J_{ATP}$, in the PncA pathway (dashed red curve) compared with the Nampt pathway (blue curve) at different temperatures. (**E**) The NAD production flux, $J_{NAD}$, in the PncA pathway compared with that in the Nampt pathway at different temperatures. (**F**) The ratio of ATP consumption to NAD production at different temperatures in PncA and Nampt pathways.

For the given optimisation problem, we found that multiple combinations of enzyme abundances could yield the desired steady-state concentration of NAD while achieving the lowest possible $J_{ATP}/J_{NAD}$ ratio. Therefore, we repeated the optimization 1000 times and analysed the distribution of enzyme abundances. The optimisation results show that the optimum was found when the amount of either PncA or Nampt was very small in comparison to the other enzymes (cf. Figure 4A). This observation indicates that under optimal conditions, only one of the two pathways is active. Consequently, it appears reasonable that bacteria typically only have either PncA or Nampt but rarely both. We used this behaviour to classify the results of the modelling into subsets with different enzyme abundances, where *Nampt* << *PncA* reflects model solutions optimised for the PncA pathway and vice versa. This enabled us to analyse the distribution of the overall enzyme abundances that facilitate NAD biosynthesis via the PncA and Nampt pathways (Figure 4B,C). Based on the simulation results, both *J_ATP_* and *J_NAD_* increased with the increase in temperature (Figure 4D,E). The ratio of ATP consumption to NAD production of the PncA pathway was always higher than that of the Nampt pathway (see Figure 4F). We used metabolic control analysis [14,15] to investigate the control exerted by metabolic enzymes on the steady-state concentrations of pathway intermediates at different temperatures. PncA had a high positive control coefficient on NAD concentration and a strong negative control coefficient on Nam concentrations at all temperatures, whereas Nampt had a positive concentration control coefficient for both Nam and NAD. See Supplementary Figures S1 and S2 for details. The steady-state concentrations of the metabolic intermediates of NAD biosynthesis via the PncA and Nampt pathways are shown in Supplementary Figure S3.

### 2.4. Optimal Pathway Performance Achieved with Mutually Exclusive Presence of PncA and Nampt

As we do not know what the bacterial NAD biosynthesis is optimized for in nature, we analysed two alternative objectives: (1) minimization of ATP consumption and (2) maximization of NAD concentration. For both scenarios, we optimized the abundance of the metabolic enzymes of NAD biosynthesis for the desired objective. Optimization was performed as described earlier. To minimize ATP consumption, we formulated the optimization problem as follows:

(4) $$\underset{E\in \left[1^{-10},100\right]}{min}\;J_{ATP}\;subject\;to:\;0.1<C_{NAD}<0.3\;B=\left\{x\vee 0.01\leq x\leq 100\right\}\;\underset{i}{\sum }E\leq 1000;i=1,\ldots ,n$$

**Figure 5:**
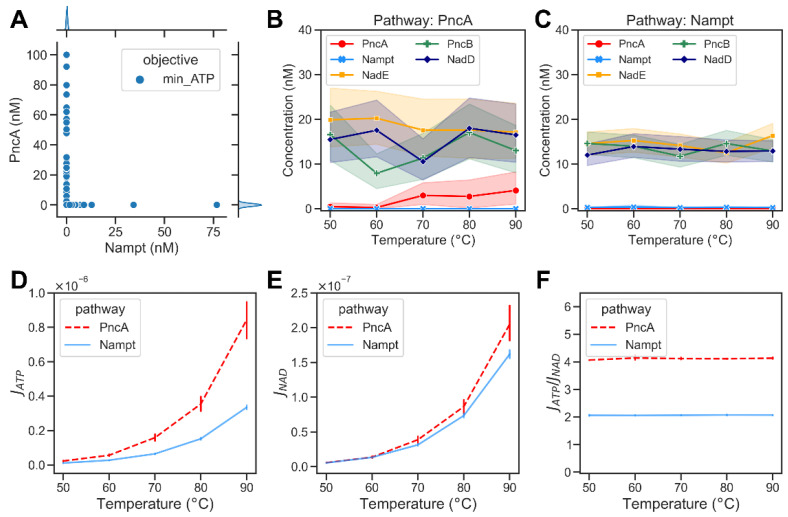
Optimization results of minimizing the total flux of ATP-consuming reactions of bacterial NAD biosynthetic pathways. (**A**) A scatter plot showing the abundances of Nampt versus PncA when minimizing the ATP consumption required to maintain the required NAD steady-state concentration ($0.1\;\mathrm{mM}<C_{NAD}<0.3\;\mathrm{mM}$). Each dot represents an optimisation result. The marginal plots show kernel density estimate plots that show the enzyme abundance distributions in the optimisation results. (**B**) Optimized abundance of enzymes at different temperatures in PncA pathway. (**C**) Optimized abundance of enzymes from Nampt pathway. (**D**) The ATP consumption flux, $J_{ATP}$, in PncA (dashed red curve) and Nampt (blue curve) pathways at different temperatures. (**E**) Computed NAD production flux, $J_{NAD}$, in PncA and Nampt pathways at different temperatures. (**F**) Calculated ratio of ATP consumption flux to NAD production flux at different temperatures in PncA and Nampt pathways.

This represents the value of the argument E (E=A∪B, where $A=\{E_{PNCA},E_{NAMPT}\}$ and $B=\left\{E_{PNCB},E_{NADD},E_{NADR},E_{PNP},E_{NADE},E_{NCE},E_{SURE}\right\}$) in the interval [1^−10^, 100] nM that minimizes objective function *J_ATP_*, with the added constraints that the steady-state concentration of NAD is between 0.1 and 0.3 mM,; the subset B is the set of all elements x, such that x is in the interval [0.01, 100] nM; and the sum of concentrations of n metabolic enzymes is less than 1000 nM. As with the previous optimization, we found that several different combinations of enzyme abundances could yield the same optimal output, and most solutions had low abundance of either the PncA or Nampt enzyme. In the PncA pathway (Nampt << PncA), NadE is the enzyme that directly affects NAD production. Thus, a higher concentration of NadE with the increase in temperature was observed, compensating the rapid hydrolysis of NAD at high temperatures. Additionally, increased abundance of PncB appeared to be important to efficiently replenish NAD. It should be noted that the substrate of PncB is NA, which, in turn, is the product of the non-enzymatic hydrolysis reactions. Thus, increasing the abundance of PncB helped to channel the flux towards the production of NAD (Figure 5B). In addition, a high abundance of NadD appeared to be advantageous. The latter was also observed for the Nampt pathway. A further analysis of the pathway fluxes showed that despite the optimal distribution of metabolic enzymes in the PncA pathway, the NAD metabolism did still require more ATP than the Nampt pathway even at high temperatures (cf. Figure 5D–F). The model-simulated steady-state concentrations of the metabolic intermediates of NAD biosynthesis via the PncA and Nampt pathways are shown in Supplementary Figure S3.

**Figure 6:**
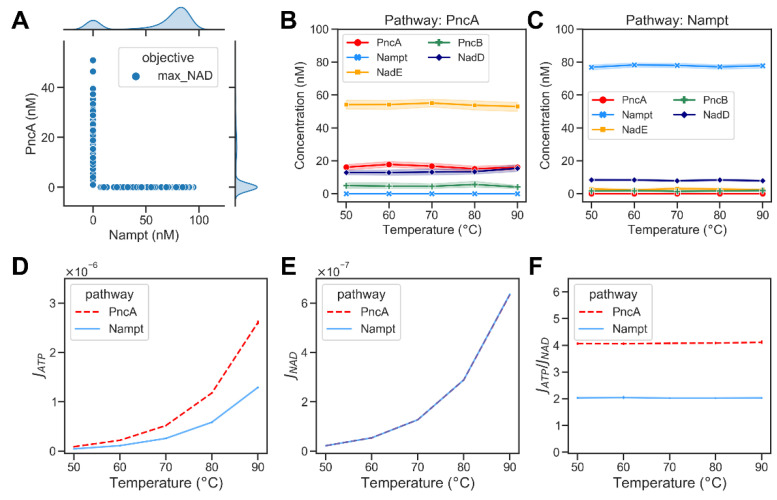
Optimization results of maximizing the NAD concentration of bacterial NAD biosynthetic pathways. (**A**) A scatter plot showing the abundances of Nampt versus PncA when maximizing the NAD production ratio. Each dot represents an optimisation result. The marginal plots show kernel density estimate plots that show the enzyme abundance distributions in the optimisation results. (**B**) Optimized abundance of enzymes at different temperatures in PncA pathway. (**C**) Optimized abundance of enzymes from Nampt pathway. (**D**) The ATP consumption flux, $J_{ATP}$, in PncA (dashed red curve) and Nampt (blue curve) pathway at different temperature (**E**) The NAD production flux, $J_{NAD}$, in PncA and Nampt pathways at different temperatures. (**F**) The ratio of ATP consumption flux to NAD production flux at different temperatures in PncA and Nampt pathways.

We then maximized the NAD concentration in our models, using the following objective function:

(5) $$\underset{E\in \left[1^{-10},100\right]}{max}\;C_{NAD}\;subject\;to:\;B=\left\{x\vee 0.01\leq x\leq 100\right\}\;\underset{i}{\sum }E\leq 1000;i=1,\ldots ,n$$

where the argument E (E=A∪B, where $A=\{E_{PNCA},E_{NAMPT}\}$ and $B=\left\{E_{PNCB},E_{NADD},E_{NADR},E_{PNP},E_{NADE},E_{NCE},E_{SURE}\right\}$) in the interval [1^−10^, 100] nM maximizes the NAD concentration (*C_NAD_*), with the added constraints that the sum of concentrations of n metabolic enzymes is less than 1000 nM and the subset B is the set of all elements x, such that x is in the interval [0.01, 100] nM. We again predominantly found solutions with low abundance of either PncA or Nampt. The PncA pathway exhibited an optimal enzyme combination with a high abundance of NadE (cf. Figure 6A). This can be understood intuitively, as the system tries to compensate for the loss of NAD through high glycohydrolysis, especially at high temperatures. The ATP consumption flux in the PncA pathway was higher than that in the Nampt pathway at different temperatures, while the NAD production flux in both pathways was similar (Figure 6C,D). Consequently, the PncA pathway was found to consume more ATP per NAD produced at all temperatures (Figure 6E).

## 3. Discussion

The enigmatic, scattered distribution of different NAD pathways in bacteria has been a matter of several previous investigations. With more than 6000 bacterial genomes available to date, we are now able to obtain a more detailed insight into the pathway distribution and its potential evolution. Our comprehensive phylogenetic analysis suggests an early evolutionary selection of either the PncA or the Nampt pathway with the respective common ancestors having both enzymes. As the PncA pathway is energetically less efficient, it is surprising that we found it in more than 50% of the analysed bacteria. We, therefore, looked at a potential role of bacterial habitats in selection preferences. This is, however, difficult, as (1) habitat information is limited and (2) habitats can change, especially over the long evolutionary timeframe we investigated. We did, however, see a predominance of the PncA pathway in extremophile organisms, indicating a potential advantage under these conditions. The measurement of the chemical stability of NAD pathway intermediates at high temperatures supported this hypothesis, as the nicotinic acid intermediates of the PncA pathway were much more stable than the nicotinamide intermediates of the Nampt pathway.

Mathematical modelling approaches allowed us to analyse hypothetical scenarios such as the dynamics of the two alternative pathways at different temperatures. We, therefore, used our ODE-based kinetic model of NAD biosynthesis to simulate temperature-dependent changes in enzyme-catalysed reaction rates. We, unfortunately, do not know what organisms are optimized for in nature; we, therefore, analysed three different objectives, i.e., (1) minimizing the ratio of ATP consumption to NAD production, (2) minimizing overall ATP consumption and (3) maximizing free-NAD concentrations. Interestingly, we found that independently of the objective function, optimization did predominantly result in models having either the PncA pathway or the Nampt pathway but rarely both. This indicates that there are no advantages in having both pathways, supporting both the early evolutionary selection and the disappearance of early ancestors that had both pathways. However, despite the higher glycohydrolysis rate of Nampt pathway intermediates, our modelling approach was not able to find any energetical advantage of the PncA pathway, as the ATP consumption was higher than that of the Nampt pathway at all temperatures. There are, of course, many parameters that we did not consider in our modelling approach, such as the thermostability of the enzymes, the achievable temperature optima and the exact activation energy for the different enzymatic reactions. Analysing the contribution of these parameters is difficult as very limited experimental data are available, leading to an unconstrained model that enables all possible scenarios (not shown). It is, furthermore, important to note that we forced the model to maintain a certain NAD concentration, while it has been shown that the high hydrolysis rates of NAD at high temperatures can impose a problem for thermophiles and hyperthermophiles [16]. Given the high stability of NAAD, it appears that a likely strategy could be to rather produce NAD on demand and thus regulate NAD production through the regulation of NadE. Unfortunately, there are very limited expression data available for thermophilic organisms at different temperatures that could support this hypothesis. We did not consider potential contributions by the de novo synthesis from aspartate either, which could compensate for the high hydrolysis of pathway intermediates. Thus, further investigations are required to better understand how extremophile organisms maintain NAD synthesis despite the high non-enzymatic hydrolysis of NAD and its intermediates.

## 4. Materials and Methods

### 4.1. Phylogenetic Analysis of Enzyme Distributions

We constructed a comprehensive overview of the taxonomic distributions of our target enzymes by performing query searches of our enzymes against the National Center for Biotechnology Information (NCBI) non-redundant protein database (nr) and subsequently combining and remapping them to the general taxonomic tree as constructed in NCBI. More specifically, we used functionally verified protein sequences of PncA and Nampt from several model organisms (see Supplementary Table S1), using their protein sequences as queries via Blastp [17]. The total number of hits returned per query was limited to 5000 sequences, and the alignment parameters followed the defaults: BLOSUM62 substitution matrix; word size, 6; gap open penalty, 11; and gap extension, 1, which was used to ensure stringent sequence selection and to avoid the inclusion of functional related but non-homologous sequences. We additionally filtered each set of resultant sequences based on cut-offs for protein length and e-value to optimize for sequence coverage and hit significance. The protein length cut-off was determined by a consensus of the hit length as taken from a histogram of all hit lengths, and the e-value cut-off was chosen as the lowest e-value at which there were sequence cross hits between our various resultant sequence sets. The cut-off values per enzyme are given in the Supplementary Table S1. The aim of sequence filtering through these cut-offs was to eliminate false positive hits across our query protein set. Taxonomic information per sequence was taken directly from the Blast results (XML2 format, taxid field). This methodology has been used previously [1].

### 4.2. Thermostability of NAD and Related Metabolites Determined by ^1^H-NMR

The compounds were dissolved in NMR buffer (25 mM sodium phosphate, pH 5.8, and 5% (*v*/*v*) D_2_O) and diluted to a final concentration of 500 µM. The samples were incubated at the indicated temperature for 10 min and chilled on ice for 5 min before measurement. NMR data were collected on a Bruker Ascend 850 MHz instrument fitted with a cryogenically cooled triple-resonance 5 mm TCI probe with pulsed-field gradients along the z-axis at 23 °C. The samples were measured by ^1^H-NMR using the pulse sequence zgesgppe, allowing water suppression to be achieved using excitation sculpting with pulsed-field gradients and perfect echo. The spectra were acquired with 64 scans and a recovery delay of 3.9 s. The spectra were assigned using standard correlation methods. Resonances of interest were integrated using the program Dynamics Center 2.5 (Bruker; Bremen Germany) and compared to baseline spectra.

### 4.3. Rate of Temperature-Dependent Non-Enzymatic Hydrolysis

Based on experimentally measured non-enzymatic hydrolysis rates (see Table 1), pre-exponential factor *A* and activation energy *E_a_* were inferred by fitting the data to the Arrhenius relation [18]:

(6) $$k=Ae^{\frac{E_{a}}{RT}}.\;$$

Using the values for *A* and *E_a_*, rate constant *k* could be calculated in dependence of absolute temperature *T* [10].

### 4.4. Mathematical Modelling of NAD Metabolism in Bacteria

For the simulation of NAD pathway dynamics, we consider a dynamic system *C* of *n* variables, which is defined as:

(7) $$\frac{dC}{dt}=f\left(C,k\right),\;$$

where reaction rates are denoted by parameter *k*. The temperature dependence of the rate laws is modelled using the Arrhenius equation (Equation (6)) as described earlier [10]. The complete list of ordinary differential equations that constitute the dynamic system of model variables are given in Supplementary Materials (cf. Equations (S1)–(S8)). The model described here uses a subset of reactions (see Figure 1), and the corresponding kinetic constants are taken from enzyme database BRENDA [19] and additionally evaluated by checking the original literature for comparable measurement conditions. Parameter values were used as in our previously published model of NAD metabolism (BioModels: MODEL1905220001; [1]). For details, see supplementary text. The initial concentration of free NAD was set to 0.3 mM, corresponding to experimentally measured free-NAD concentrations. The initial concentration of all other pathway intermediates was set to 0, unless stated otherwise. The model did converge on the same steady state as long as the sum of the concentration of all Nam-containing intermediates remained 0.3 mM. Influx and growth were neglected in the simulations to reduce the complexity and number of parameters. All parameters were assumed to hold for base temperature *T*_0_ *=* 37.5 °C.

### 4.5. Software and Data

The temperature-dependent model of NAD metabolism was submitted to the BioModels database (model MODEL2103290001). The ETE3 toolkit v3.1.2 [20] was used to visualize the phylogenetic trees. The python scripts used to perform the phylogenetic analysis are available on GitHub: https://github.com/MolecularBioinformatics/Phylogenetic-analysis (accessed on 25 March 2019). The steady-state calculation of fluxes and concentrations was performed using COPASI 4.29 [11]. The Python scripts used to calculate the modelling results of this paper can be downloaded from GitHub: https://github.com/MolecularBioinformatics/thermophilesNAD (accessed on 12 May 2022).

## Figures and Tables

**Table 1:** Measured hydrolysis rates (%/min) at different temperatures (°C) and calculated pre-exponential factor *A* and activation energy *E_a_* based on the fitting of the data using the Arrhenius equation (Equation (1)).

Compound	Temperature (°C)	Hydrolysis Rate (%/min)	Prefactor *A*	Activation Energy *E_a_* (KJ/mol)
NR	50	0.68 ± 0.04	27083.62	72.41
	75	2.06 ± 0.10		
	90	6.26 ± 0.07		
NMN	50	0.07 ± 0.02	340657.92	82.40
	75	0.62 ± 0.03		
	90	2.76 ± 0.13		
NAD	50	0.00 ± 0.01	1.37 × 10^11^	123.16
	75	0.45 ± 0.01		
	90	1.60 ± 0.05		
NAR	50	0.01 ± 0.01	1.25 × 10^13^	138.60
	75	0.12 ± 0.02		
	90	0.86 ± 0.02		
NAMN	50	0.00 ± 0.01	2.12 × 10^13^	142.49
	75	0.05 ± 0.03		
	90	0.40 ± 0.01		

## Data Availability

The model file can be downloaded from BioModels: MODEL2103290001. The Python scripts used to calculate the results shown in this paper can be downloaded from Github: https://github.com/MolecularBioinformatics/thermophilesNAD (accessed on 25 March 2022) and https://github.com/MolecularBioinformatics/Phylogenetic-analysis (accessed on 10 July 2019).

## Supplementary Materials

The following supporting information can be downloaded at: https://www.mdpi.com/article/10.3390/metabo12070569/s1, Figure S1: Concentration control coefficients of NAD biosynthesis with exclusive presence of PncA enzyme. Each subplot shows the concentration control coefficient on the metabolic intermediates of PncA pathway due to the perturbation in the enzyme concentration corresponding to an enzyme catalyzed reaction (y-axis) at different temperatures (x-axis). Refer the computational model submitted at the BioModels database as MODEL2103290001 for details about the reactions; Figure S2: Concentration control coefficients of NAD biosynthesis via exclusive presence of Nampt enzyme. Each subplot shows the concentration control coefficient on the metabolic intermediates of Nampt pathway due to the perturbation in the enzyme concentration corresponding to an enzyme catalyzed reaction (y-axis) at different temperatures (x-axis). Refer the computational model submitted at the BioModels database as MODEL2103290001 for details about the reactions; Figure S3: Simulated steady-state concentrations of metabolic intermediates of NAD biosynthesis via PNCA and NAMPT pathway at different temperatures using the mathematical model of NAD biosynthesis. Model-simulated concentrations of pathway intermediates while A) minimization of the ratio of ATP consumption (J_ATP_) to NAD production flux (J_NAD_), B) minimization of the ATP consumption flux (J_ATP_), and C) maximization of the free NAD concentration (C_NAD_). Error bars show the different simulated concentrations due to different combinations of enzyme concentrations found as a solution to the optimization problem at a given temperatures; Table S1: Seed sequences for phylogenetic analysis: enzyme identity and cutoffs for minimum length and maximum E-value; Table S2: An overview of rate laws used by the metabolic enzymes of NAD pathway. Ref. [21] is cited in the Supplementary Materials file.
