# Supplementary material for: Early Evolutionary Selection of NAD Biosynthesis Pathway in Bacteria
Source: Metabolites. 2022 Jun 21;12(7):569. doi: 10.3390/metabo12070569 (PMC9316036; doi:10.3390/metabo12070569)
Supplement: Supplementary file 1 [file metabolites-12-00569-s001.zip › metabolites-1747283-Supplementary Updated.pdf]

metabolites

MDPI

Supplementary Text

Early evolutionary selection of NAD biosynthesis pathway in bacteria

Suraj Sharma [1], Yin-Chen Hsieh [1], Jörn Dietze [1], Mathias Bockwoldt [2], Øyvind Strømland [3], Mathias Ziegler [3] and Ines Heiland [1, 4*]

1. Seed sequences for phylogenetic analysis

Supplementary Table S1: Seed sequences for phylogenetic analysis: enzyme identity and cutoffs for minimum length and maximum E-value.

Enzyme	Gene	Full Name	UniProt ID	Species	Min. Length	E-value
PncA	NIC1	Nicotinamidase 1	Q8S8F9	*A. thaliana*	170	1e^{-30}
PncA	Pnc-1	Isochorismatase domain-containing protein	Q9N426	*C. elegans*	170	1e^{-30}
PncA	pncA	Nicotinamidase	P21369	*E. coli*	170	1e^{-30}
PncA	PNC1	Nicotinamidase	P53184	*S. cerevisiae*	170	1e^{-30}
Nampt	NPT1	Nicotinamide phosphoribosyl-transferase	D9I2J1	*C. reinhardtii*	400	1e^{-30}
Nampt	NAMPT	Nicotinamide phosphoribosyl-transferase	P43490	*H. sapiens*	400	1e^{-30}
Nampt	Slr0788	Pre-B cell enhancing factor	Q55929	*Synechocystis sp.*	400	1e^{-30}

2. Dynamic model of NAD metabolism in bacteria

Our dynamic model of NAD metabolism via PNCA and NAMPT pathway is composed of the following ordinary differential equations that can simulate temperature-dependent steady states of pathway intermediates $C_i(i = 1, \dots, n)$:

$$\frac{d[NA]}{dt} = V \cdot (v_{PNCA} - v_{PNCB} + v_{PNP} + v_{NAMN}^T + v_{NADR}^T) \quad (S1)$$

$$\frac{d[NAMN]}{dt} = V \cdot (v_{PNCB} - v_{NT5} + v_{NADR} + v_{NADD} - v_{NAMN}^T) \quad (S2)$$

$$\frac{d[NAAD]}{dt} = V \cdot (v_{NADD} - v_{NADE}) \quad (S3)$$

$$\frac{d[NAD]}{dt} = V \cdot (v_{NADE} + v_{NADD} - v_{SIRT} - v_{NAD}^T) \quad (S4)$$

$$\frac{d[NMN]}{dt} = V \cdot (v_{NADD} + v_{NAMPT} + v_{NADR} - v_{NT5} - v_{NMN}^T) \quad (S5)$$

$$\frac{d[Nam]}{dt} = V \cdot (v_{SIRT} + v_{PNP} - v_{NAMPT} - v_{PNCA} + v_{NAD}^T + v_{NR}^T + v_{NMN}^T) \quad (S6)$$

$$\frac{d[NR]}{dt} = V \cdot (v_{NT5} - v_{PNP} - v_{NADR} - v_{NR}^{T}) \qquad (S7)$$

$$\frac{d[NAR]}{dt} = V \cdot (v_{NT5} - v_{PNP} - v_{NADR} - v_{NAR}^{T}) \qquad (S8)$$

Here, V denotes the bacterial cell volume, while $v_j (j = 1, \ldots, m;\ m \neq n)$ and $v_l^T (l = 1, \ldots, p;\ p \leq n)$ represent the rate of jth enzymatic reaction and thermolysis of lth intermediate, respectively. The jth flux can be defined as $J_j = V \cdot v_j$. The corresponding kinetic constants are taken from the enzyme database BRENDA and additionally evaluated by checking the original literature for comparable measurement conditions. For details about other kinetic parameters see the computational model submitted at the BioModels database as MODEL2103290001.

3. Rate laws

Supplementary Table S2: an overview of rate laws used by the metabolic enzymes of NAD pathway.

Enzyme	EC number	Rate law
PncA	3.5.1.19	Product Inhibition
PncB	2.4.2.11	MM Irreversible
NadD	2.7.7.1	Substrate Competition
NadE	6.3.5.1	MM Irreversible
Nampt	2.4.2.12	Competitive Inhibition
NCE	3.5.1.-	Product Inhibition
SurE	3.1.3.5	MM Irreversible
PNP	2.4.2.1	MM Irreversible
NadR	2.7.1.173	MM Irreversible

Michaelis-Menten (MM) Irreversible:

$$v = \frac{E_{total} \cdot k_{cat} \cdot S}{K_m + S} \qquad (S9)$$

Product Inhibition:

$$v = \frac{E_{total} \cdot k_{cat} \cdot S}{K_m + S + \frac{K_m \cdot P}{K_{i,P}}} \qquad (S10)$$

Competitive Inhibition:

$$v = \frac{E_{total} \cdot k_{cat} \cdot S}{K_m + S + \frac{K_m \cdot I}{K_{i,I}}} \qquad (S11)$$

Substrate Competition:

$$v = \frac{E_{total} \cdot \frac{k_{cat} \cdot A \cdot B}{K_{m,A}} - \frac{k_{cat} \cdot P \cdot Q}{K_{m,P}}}{1 + \frac{A}{K_{m,A}} + \frac{B}{K_{m,B}} + \frac{P}{K_{m,P}} + \frac{Q}{K_{m,Q}}} \qquad (S12)$$

Thermolysis Rate:

$$v^T = k^T \cdot S \qquad (S13)$$

Where the thermolysis rate constant k^T at a temperature T is calculated using Arrhenius relation as

$$k^T = Ae^{-\frac{E_a}{R\cdot T}}. \qquad (S14)$$

The temperature dependence of the rate constant (k_{cat}) at temperature T can be calculated using the method described by [10] as

$$k_{cat}(T) = \hat{k}_{cat} \cdot e^{-\frac{E_a}{R}\cdot\frac{(T-T_0)}{T\cdot T_0}} \qquad (S15)$$

where $\hat{k}_{cat}$ is rate constant of enzyme-catalysed reaction at base temperature T_0.

4. Metabolic Control Analysis

Metabolic control analysis (MCA) is a mathematical framework that quantifies how fluxes and concentrations depend on kinetic parameters such as enzyme concentrations (cf. [14,21]). The system properties that are at the centre of MCA theory are the control coefficients. In response to a relative change in a kinetic parameter (p_k), control coefficient measures the relative steady state change in pathway flux (J_j) or metabolite concentration (C_i), known as flux control coefficients and concentration control coefficients, respectively.

To study the control exerted by an enzyme catalysing reaction j on the steady-state concentrations of metabolites (C_i), we used the metabolic control analysis method in COPASI 4.29 [13] to calculate the concentration control coefficients describing the effect of perturbation in the enzyme concentration. Further, we analysed the concentration control coefficients yielded by the enzyme distributions with mutually exclusive presence of PncA and Nampt enzyme to understand the control exerted on steady-state concentrations of metabolites (see Figure S1 and S2, respectively). It clearly shows i) the varying control exerted by different enzymes on the steady-state concentrations between PncA and Nampt pathway, and ii) different enzymes exert varying control on the steady-state concentration of metabolites at different temperatures.

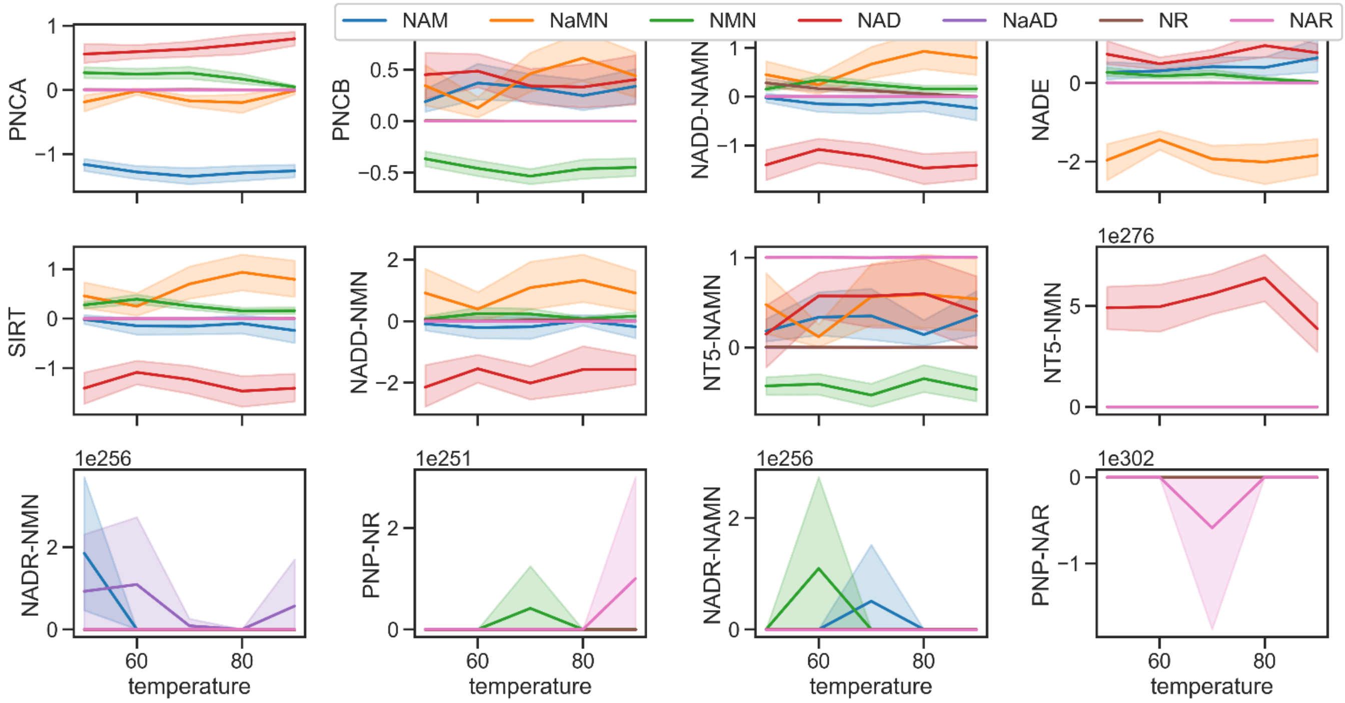

Supplementary Figure S1. Concentration control coefficients of NAD biosynthesis with exclusive presence of PncA enzyme. Each subplot shows the concentration control coefficient on the metabolic intermediates of PncA pathway due to the perturbation in the enzyme concentration corresponding to an enzyme catalyzed reaction (y-axis) at different temperatures (x-axis). Refer the computational model submitted at the BioModels database as MODEL2103290001 for details about the reactions.

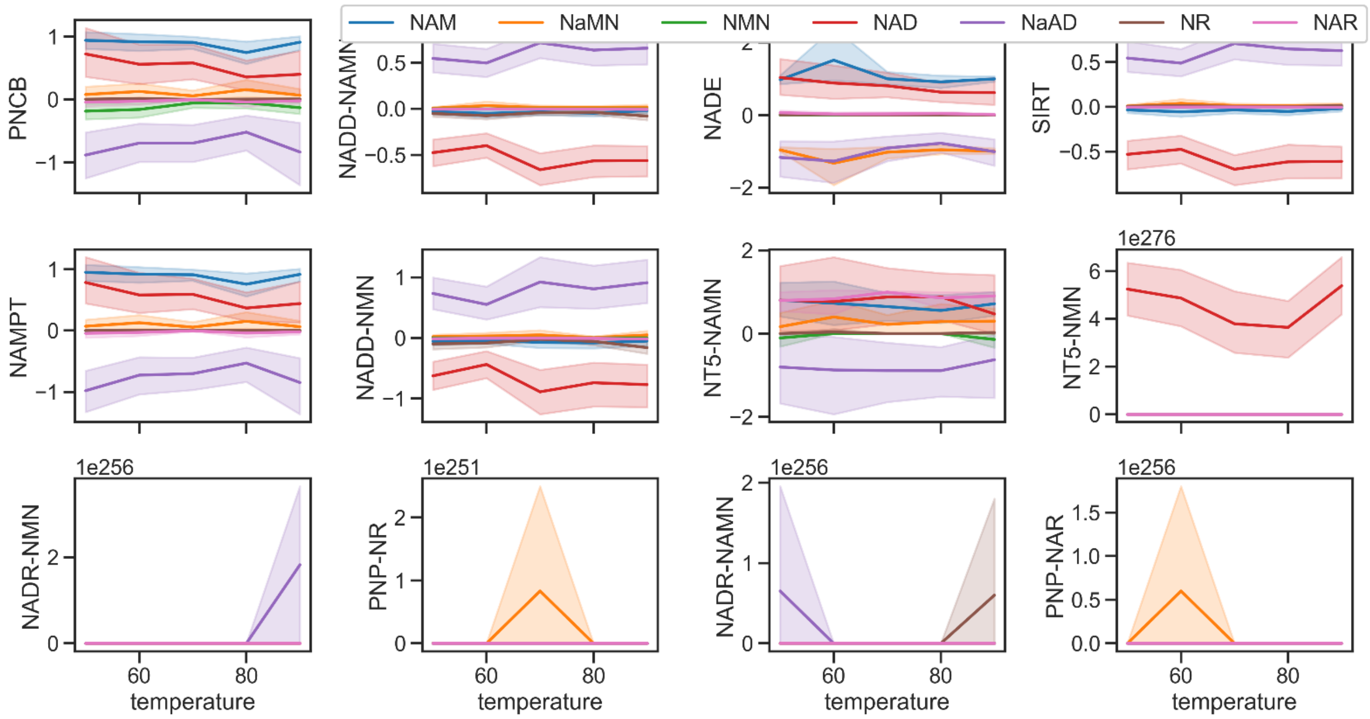

Supplementary Figure S2. Concentration control coefficients of NAD biosynthesis via exclusive presence of Nampt enzyme. Each subplot shows the concentration control coefficient on the metabolic intermediates of Nampt pathway due to the perturbation in the enzyme concentration corresponding to an enzyme catalyzed reaction (y-axis) at different temperatures (x-axis). Refer the computational model submitted at the BioModels database as MODEL2103290001 for details about the reactions.

5. Steady-state concentrations of metabolites of PNCA and NAMPT pathway

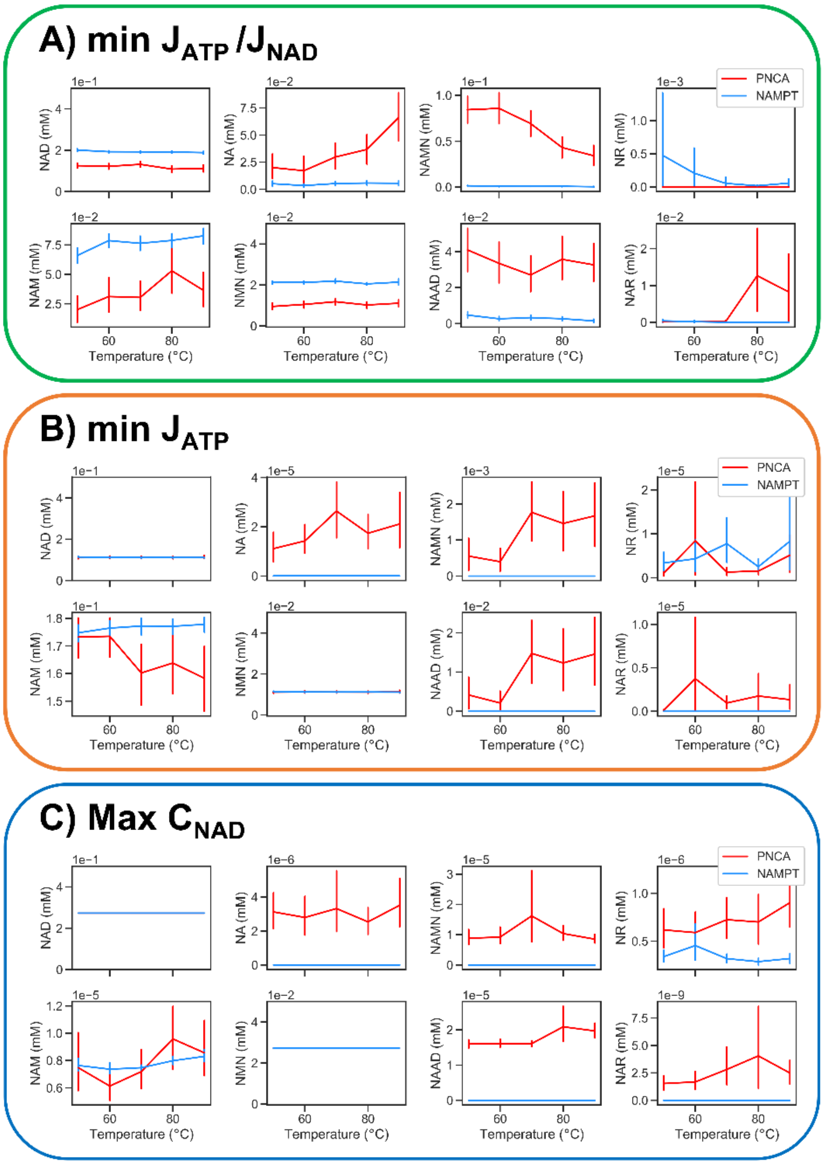

Supplementary Figure S3. Simulated steady-state concentrations of metabolic intermediates of NAD biosynthesis via PNCA and NAMPT pathway at different temperatures using the mathematical model of NAD biosynthesis. Model-simulated concentrations of pathway intermediates while A) minimization of the ratio of ATP consumption (J_{ATP}) to NAD production flux (J_{NAD}), B) minimization of the ATP consumption flux (J_{ATP}), and C) maximization of the free NAD concentration (C_{NAD}). Error bars show the different simulated concentrations due to different combinations of enzyme concentrations found as a solution to the optimization problem at a given temperatures.

References

[10] Bodenstein, C.; Heiland, I.; Schuster, S. Calculating activation energies for temperature compensation in circadian rhythms. *Phys. Biol.* **2011**, *8*, 056007. https://doi.org/10.1088/1478-3975/8/5/056007.

[13] Hoops, S.; Sahle, S.; Gauges, R.; Lee, C.; Pahle, J.; Simus, N.; Singhal, M.; Xu, L.; Mendes, P.; Kummer, U. COPASI—A COmplex PAthway SImulator. *Bioinformatics* **2006**, *22*, 3067–3074. https://doi.org/10.1093/bioinformatics/btl485.

[14] Heinrich, R.; Rapoport, T.A. A Linear Steady-State Treatment of Enzymatic Chains. *Eur. J. Biochem.* **1974**, *42*, 89–95. https://doi.org/10.1111/j.1432-1033.1974.tb03318.x.

[21] Kacser, H.; Burns, J.A. The control of flux. *Symp. Soc. Exp. Biol.* **1973**, 27, 65–104. Available online: http://www.ncbi.nlm.nih.gov/pubmed/4148886 (accessed on 17 May 2017).
